# Diabetes Mellitus, Microalbuminuria, and Subclinical Cardiac Disease: Identification and Monitoring of Individuals at Risk of Heart Failure

**DOI:** 10.1161/JAHA.117.005539

**Published:** 2017-07-17

**Authors:** Peter P. Swoboda, Adam K. McDiarmid, Bara Erhayiem, David P. Ripley, Laura E. Dobson, Pankaj Garg, Tarique A. Musa, Klaus K. Witte, Mark T. Kearney, Julian H. Barth, Ramzi Ajjan, John P. Greenwood, Sven Plein

**Affiliations:** ^1^ Multidisciplinary Cardiovascular Research Centre & Division of Biomedical Imaging Leeds Institute of Cardiovascular and Metabolic Medicine University of Leeds United Kingdom; ^2^ Leeds Teaching Hospitals NHS Trust Leeds United Kingdom

**Keywords:** biomarker, cardiac magnetic resonance imaging, diabetes mellitus, diastolic dysfunction, echocardiography, extracellular volume fraction, renin angiotensin system, Metabolic Syndrome, Heart Failure, Magnetic Resonance Imaging (MRI), Echocardiography, Diagnostic Testing

## Abstract

**Background:**

Patients with type 2 diabetes mellitus and elevated urinary albumin:creatinine ratio (ACR) have increased risk of heart failure. We hypothesized this was because of cardiac tissue changes rather than silent coronary artery disease.

**Methods and Results:**

In a case‐controlled observational study 130 subjects including 50 ACR+ve diabetes mellitus patients with persistent microalbuminuria (ACR >2.5 mg/mol in males and >3.5 mg/mol in females, ≥2 measurements, no previous renin–angiotensin–aldosterone therapy, 50 ACR−ve diabetes mellitus patients and 30 controls underwent cardiovascular magnetic resonance for investigation of myocardial fibrosis, ischemia and infarction, and echocardiography. Thirty ACR+ve patients underwent further testing after 1‐year treatment with renin–angiotensin–aldosterone blockade. Cardiac extracellular volume fraction, a measure of diffuse fibrosis, was higher in diabetes mellitus patients than controls (26.1±3.4% and 23.3±3.0% *P*=0.0002) and in ACR+ve than ACR−ve diabetes mellitus patients (27.2±4.1% versus 25.1±2.9%, *P*=0.004). ACR+ve patients also had lower E′ measured by echocardiography (8.2±1.9 cm/s versus 8.9±1.9 cm/s, *P*=0.04) and elevated high‐sensitivity cardiac troponin T 18% versus 4% ≥14 ng/L (*P*=0.05). Rate of silent myocardial ischemia or infarction were not influenced by ACR status. Renin–angiotensin–aldosterone blockade was associated with increased left ventricular ejection fraction (59.3±7.8 to 61.5±8.7%, *P*=0.03) and decreased extracellular volume fraction (26.5±3.6 to 25.2±3.1, *P*=0.01) but no changes in diastolic function or high‐sensitivity cardiac troponin T levels.

**Conclusions:**

Asymptomatic diabetes mellitus patients with persistent microalbuminuria have markers of diffuse cardiac fibrosis including elevated extracellular volume fraction, high‐sensitivity cardiac troponin T, and diastolic dysfunction, which may in part be reversible by renin–angiotensin–aldosterone blockade. Increased risk in these patients may be mediated by subclinical changes in tissue structure and function.

**Clinical Trial Registration:**

URL: http://www.clinicaltrials.gov. Unique identifier: NCT01970319.

**Clinical Perspective**

**What Is New?**

- Asymptomatic diabetes mellitus patients with persistent microalbuminuria have markers of diffuse cardiac fibrosis including elevated cardiac extracellular volume fraction, high‐sensitivity cardiac troponin T, and diastolic dysfunction, which may in part be reversible by renin–angiotensin–aldosterone blockade.

**What Are the Clinical Implications?**

- These findings suggest that increased risk in this patient group is mediated by subclinical changes in tissue structure and function rather than occult coronary artery disease. Future randomized studies are needed to establish whether extracellular volume fraction, high‐sensitivity cardiac troponin T, or diastolic dysfunction themselves or the associated adverse prognosis can be altered by intervention.

## Introduction

Type 2 diabetes mellitus is well established as an independent risk factor for the development of heart failure and does not appear to show a relationship with conventional risk factors including age, sex, existing coronary artery disease (CAD), and hypertension.^1^ Patients with diabetes mellitus and heart failure have a poor clinical prognosis with higher mortality than normoglycemic heart failure patients.^2^, ^3^ Epidemiological evidence suggests that poor control of blood glucose in type 2 diabetes mellitus is associated with increased rate of heart failure.^4^ However, data from randomized controlled trials demonstrate that intensive control of glucose does not reduce the risk of hospitalization for heart failure.^5^ To complicate matters, recent evidence suggests that certain hypoglycemic agents used can modulate the risk of heart failure in patients with diabetes mellitus, independently of glucose‐lowering properties.^6^, ^7^, ^8^

Patients with type 2 diabetes mellitus and albuminuria have higher risk of heart failure,^9^, ^10^ and treatment directed at modulating the renin–angiotensin–aldosterone system (RAAS) decreases rate of hospitalization with cardiac decompensation. Data from several large randomized controlled trials have shown that elevated albumin:creatinine ratio (ACR) is associated with increased risk of new‐onset heart failure.^11^ Furthermore, RAAS modulation with either an angiotensin‐converting enzyme (ACE) inhibitor or angiotensin receptor blocker decreases the risk of heart failure hospitalization.^12^, ^13^

Multiparametric cardiovascular imaging has been used to detect subclinical changes in cardiac structure and function in type 2 diabetes mellitus including increased left ventricular (LV) mass, concentric remodeling of the left ventricle,^14^, ^15^, ^16^ and diastolic dysfunction as measured by conventional Doppler and tissue Doppler echocardiography.^17^, ^18^ Cardiovascular magnetic resonance (CMR) T1 mapping studies have also suggested that type 2 diabetes mellitus is associated with diffuse fibrosis and increased cardiac extracellular volume fraction (ECV).^19^, ^20^, ^21^

In addition, several cardiovascular imaging methods have been used to show that type 2 diabetes mellitus is associated with increased rates of CAD,^22^ silent myocardial ischemia,^23^ and silent myocardial infarction (MI) .^24^, ^25^, ^26^ Patients with type 2 diabetes mellitus have elevated levels of cardiac biomarkers including high‐sensitivity cardiac troponin T (hs‐cTnT) and amino terminal B type natriuretic peptide (NT‐proBNP), which appear to be associated with adverse cardiovascular outcomes including heart failure.^27^, ^28^, ^29^

Given that the exact pathogenic mechanisms for heart failure in diabetes mellitus remain unclear, we aimed, through a combination of an observational and interventional study, to establish the pattern of subclinical cardiac disease in patients with diabetes mellitus and persistent microalbuminuria and determine whether the risk of heart failure is mediated by occult CAD or changes in cardiac structure and tissue characteristics. The interventional part of the work investigated whether detected abnormalities are reversible after blockade of the RAAS.

## Methods

### Enrollment Criteria

The study was approved by the National Research Ethics Service (13/YH/0098) and all participants gave informed consent. A total of 100 patients with type 2 diabetes mellitus were recruited from 30 primary care health centers in the local area from August 2013 to March 2015. Fifty consecutive patients with persistent microalbuminuria (ACR+ve), urinary ACR >2.5 mg/mol in males and >3.5 mg/mol in females, were recruited after their annual diabetes mellitus check before commencement on an ACE inhibitor in line with national guidelines.^30^ All ACR+ve patients had laboratory ACR repeated within 4 months to confirm persistent microalbuminuria. In addition, 50 patients with type 2 diabetes mellitus were recruited who had never had ACR above the thresholds (ACR−ve). This group was prospectively matched by frequency matching for age, sex, and clinic blood pressure to ACR+ve patients. Exclusion criteria for all subjects were known cardiac disease, kidney disease (estimated glomerular filtration rate <30 mL/min per 1.73 m^2^), uncontrolled hypertension, and treatment with insulin or ACE inhibitor/angiotensin receptor blocker. Ten‐year likelihood of developing CAD and related mortality were calculated using the Framingham risk score.^31^ In addition, 30 age‐ and sex‐matched controls with no diabetes mellitus and no known cardiovascular disease were recruited. The study was conducted in accordance with the declaration of Helsinki and all subjects gave informed written consent.

In accordance with current guidelines, all patients with persistent microalbuminuria were started on an ACE inhibitor by their primary care team following the baseline investigations^30^ and uptitrated to the maximum tolerated dose. Those who could not tolerate an ACE inhibitor because of cough were started on an angiotensin receptor blocker. All testing was repeated after 1‐year treatment with RAAS inhibition.

### Echocardiography and Blood Pressure Assessment

All patients underwent echocardiography (Vivid e9, GE Medical Systems, Milwaukee, WI) focused on Doppler measurements of mitral inflow and tissue Doppler imaging of the lateral and medial mitral annulus for the assessment of diastolic function. E/A ratio, E**′**, A**′,** and S**′** were measured using inbuilt software blinded to clinical details according to international guidelines.^32^ All patients underwent 24‐hour blood pressure monitoring with a Welch‐Allyn 6100 ambulatory blood pressure monitor.

### CMR Protocol

Patients and controls underwent CMR using an identical protocol on a dedicated cardiovascular 3 Tesla Philips Achieva system equipped with a 32‐channel coil and MultiTransmit^®^ technology. Data were acquired during breath‐holding at end expiration. T1 maps were acquired in a midventricular slice. Native T1 mapping used a breath‐held Modified Look‐Locker Inversion recovery (MOLLI) acquisition (ECG triggered 5(3s)3, spatial resolution 1.98×1.98×10 mm^3^ [reconstructed to 1.25×1.25 mm^2^]), single‐shot, SENSE factor 2, prepulse delay 350 ms, trigger delay set for end‐diastole (adaptive), flip angle 35°, acquisition duration per image 170 to 185 ms (dependent on field of view).

Next, myocardial perfusion CMR was planned in the same 3 short‐axis slices using a spoiled turbo gradient‐echo sequence. This was performed while intravenous adenosine was administered at 140 μg/kg per minute for 3 minutes and repeated >10 minutes later at rest. Gadobutrol (0.075 mmol/kg) was administered for each stress and rest perfusion imaging (Gadovist^®^, Bayer Pharma, Berlin, Germany).

Cine images covering the entire heart in the LV short axis (slice thickness 10 mm); left atrium in short axis (slice thickness 5 mm) and in orthogonal long axis planes was acquired. Late gadolinium enhancement (LGE) in matching LV short‐ and long‐axis planes was carried out more than 6 minutes after rest perfusion imaging as described previously.^26^

Postcontrast T1 mapping was carried out exactly 15 minutes following last contrast injection using the identical pulse sequence and planning as the native T1 map (above).

### CMR Interpretation

CMR data were assessed quantitatively using commercially available software (CVI42, Circle Cardiovascular Imaging Inc, Calgary, Canada). Epicardial and endocardial borders were traced offline on the LV cine stack at end‐diastole and end‐systole to calculate end‐diastolic, end‐systolic LV volumes, stroke volume, ejection fraction (EF), and LV mass. Left atrial volume was calculated by tracing the left atrial endocardial border at end‐systole when the left atrium was largest.

Pre‐ and postcontrast myocardial T1 values with a 3‐parameter exponential fit with Look‐Locker correction were measured from the midventricular short‐axis slice in the septum. ECV was calculated from native and postcontrast T1 times of myocardium and blood pool and hematocrit as previously reported excluding any areas of scar seen on LGE imaging.^33^ Perfusion imaging was interpreted independently by 2 physicians experienced in CMR for the presence of clinically significant ischemia (>1 segment of American Heart Association 16 segment model).^34^ Mass of LGE was quantified using the Otsu automated threshold method and divided by LV mass calculated from short axis cines to give a percentage of LV with LGE.^35^

### Biomarker Assessment

Twenty milliliters of blood was drawn from each subject at the time of each CMR. Full blood count, urea and electrolytes, and hemoglobin A1c were measured at that time. Serum was stored at −70°C and tested for hs‐cTnT (typical coefficient of variability 4.4% at 13.7 ng/L and 3.6% at 95.3 ng/L), NT‐proBNP (typical coefficient of variability 2.9% at 91 ng/L and 2.1% at 415 ng/L); Cobas 8000 (Roche Diagnostics, Burgess Hill, West Sussex, UK) and aldosterone (typical between batch coefficient of variability <10% at 282, 402 and 1833 pmol/L); local laboratory‐validated radioimmunoassay. Patients with hs‐cTnT ≥14 ng/L and NT‐proBNP ≥125 ng/L were defined as having elevated levels of these biomarkers that have been independently associated with adverse outcomes in type 2 diabetes mellitus.^27^, ^28^, ^29^

### Statistical Analysis and Power Calculations

Statistical analysis was performed using IBM SPSS^®^ Statistics 20.0 (IBM Corp, Armonk, NY). Continuous variables were expressed as mean±SD. Categorical variables were expressed as N (%) and compared using Fisher exact test. Shapiro–Wilk test was used to test normality and depending on the result ANOVA and and Kruskal–Wallis test were used to compare means of ACR+ve, ACR−ve, and controls. Paired *t* test or Wilcoxon test were used to compare parameters before and after treatment. *P*<0.05 was considered statistically significant.

Based on reported difference in ECV between those with and without diabetes mellitus,^21^ it was predicted that 49 patients per group were needed to detect a 2% difference in ECV between ACR+ve and ACR−ve patients and that 26 patients would be needed to detect a 2% change in ECV after RAAS inhibition (assuming SD 3%, α=0.05, power=90%).

## Results

### Patient Characteristics

**Figure 1:**
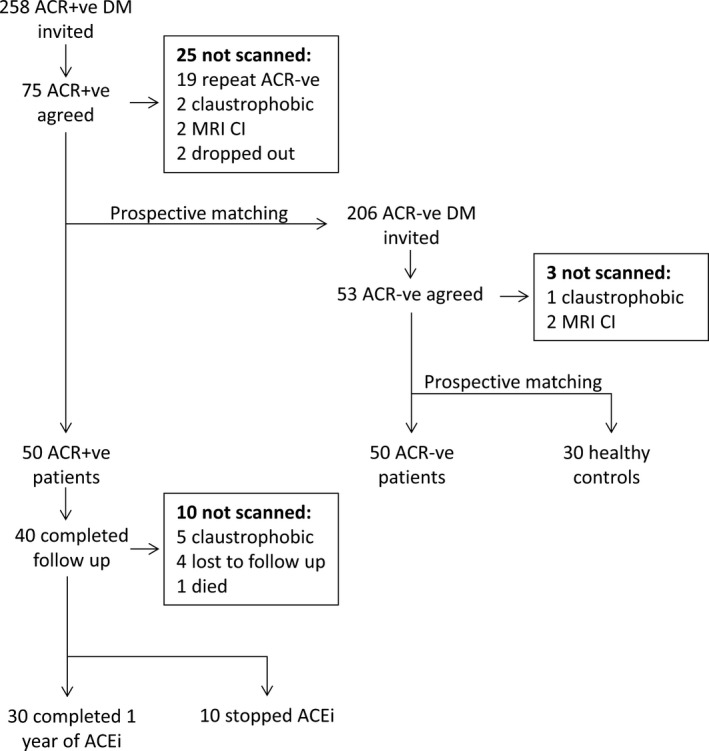
Recruitment flowchart. ACEi indicates Angiotensin converting enzyme inhibitor; ACR, albumin:creatinine ratio; ACR+ve, albumin:creatinine ratio positive; ACR−ve, albumin:creatinine ratio negative; CI, contraindication; DM, diabetes mellitus; MRI, magnetic resonance imaging.

A total of 464 patients with diabetes mellitus were approached, of whom 128 patients agreed to participate with 50 ACR+ve and 50 ACR−ve completing the full protocol (Figure 1). Six of 30 controls chose not to receive adenosine stress because of potential side effects. Baseline patient and control characteristics and medications are shown in Table 1. There was no difference between ACR+ve and ACR−ve patients in age, sex, body mass index, diabetes mellitus duration, hemoglobin A1c, or 24‐hour blood pressure.

**Table 1:** Subject Characteristics

	Control	ACR−ve	ACR+ve	*P* Value Between 3 Groups	*P* Value for ACR−ve and ACR+ve
N	30	50	50		
Age (y)	59.1±11.5	61.1±9.1	60.2±12.7	0.76	0.69
Male sex, n (%)	21 (70)	42 (84)	40 (80)	0.32	0.60
Body mass index, kg/m^2^	26.6±3.2	28.6±4.0	29.1±4.6	0.04	0.61
Duration of diabetes mellitus (y)	···	4.6±4.4	5.3±4.4	···	0.40
HbA1c, mmol/mol	36.2±3.8	60.2±13.7	65.9±23.9	<0.0001	0.47
Systolic BP, mm Hg	130±13	130±13^a^	133±17^a^	0.66	0.46
Diastolic BP, mm Hg	70±11	71±8^a^	74±10^a^	0.20	0.12
Total cholesterol	···	4.4±1.2	4.4±1.1	···	0.94
Smoking	0	6	9		0.40
Framingham 10‐y risk of coronary disease, %	···	14.6±7.0	16.5±8.2		0.21
Framingham 10‐y risk of coronary mortality, %	···	3.5±3.0	4.3±3.1		0.24
Metformin	0	49	38	<0.0001	0.002
Sulfonylurea	0	12	21	<0.0001	0.09
Gliptin	0	5	6	0.16	0.75
Other hypoglycemic	0	3	1	0.28	0.62
Insulin	0	0	0	1.0	1.0
ACE inhibitor	0	0	0	1.0	1.0
β‐Blocker	0	1	3	0.28	0.62
Calcium channel blocker	1	6	4	0.40	0.74
Diuretic	0	2	3	0.40	1.0
Statin	2	36	33	<0.0001	0.67
Aspirin	3	4	14	0.01	0.02
LV EDV, mL	157.6±42.1	149.2±32.4	147.2±36.3	0.37	0.33
LV EDV index, mL/m^2^	81.0±18.8	74.1±13.9	73.1±14.5	0.11	0.29
LV ESV, mL	65.4±25.0	58.2±15.6	59.5±23.7	0.41	0.54
Ejection fraction, %	59.1±7.9	61.2±5.1	60.6±6.9	0.21	0.60
LV mass, g	93.5±23.6	93.9±17.6	98.5±23.3	0.07	0.39
LV mass index, g/m^2^	48.0±9.9	46.8±7.7	48.9±9.4	0.06	0.35
Mass/end diastolic volume, g/mL	0.60±0.11	0.65±0.14	0.68±0.12	<0.0001	0.07
Left atrial volume, mL	91.6±26.6	87.5±17.0	89.8±22.5	0.87	0.83
Left atrial volume index, mL/m^2^	46.9±12.3	43.7±8.2	44.9±9	0.56	0.58
Native T1, ms	1210±47	1232±36	1253±66	0.002	0.05^b^
Extracellular volume, %	23.3±3.0	25.1±2.9	27.2±4.1	<0.0001	0.004^b^
Myocardial infarction, n (%)	0 (0)	8 (16)	9 (18)	0.01^c^	0.79
Mass of infarction, g	···	3.1±2.1	8.7±11.7	···	0.50
Serum aldosterone, pmol/L	···	299±195	323±183	···	0.32
hs‐cTnT ≥14 ng/L, n (%)	···	2 (4)	9 (18)		0.05^b^
NT‐proBNP ≥125 ng/L, n (%)	···	4 (8)	7 (14)		0.52
E/A ratio	···	0.87±0.27	0.85±0.34		0.69
E**′** average, cm/s	···	8.9±1.9	8.2±1.9		0.04^b^
E/E**′** average	···	7.1±2.3	7.2±2.0		0.67
S**′** average, cm/s	···	9.7±2.0	9.4±1.8		0.45

ACE indicates angiotensin‐converting enzyme; ACR, albumin:creatinine ratio; EDV, end diastolic volume; ESV, end systolic volume; HbA1c, hemoglobin A1c; hs‐cTnT, high‐sensitivity cardiac troponin T; LV, left ventricle; NT‐proBNP, amino terminal B type natriuretic peptide.

a Twenty‐four‐hour BP.

b *P*≤0.05.

c Diabetes mellitus and control.

### Effect of Diabetes Mellitus and Microalbuminuria on Cardiac Structure and Composition

**Figure 2:**
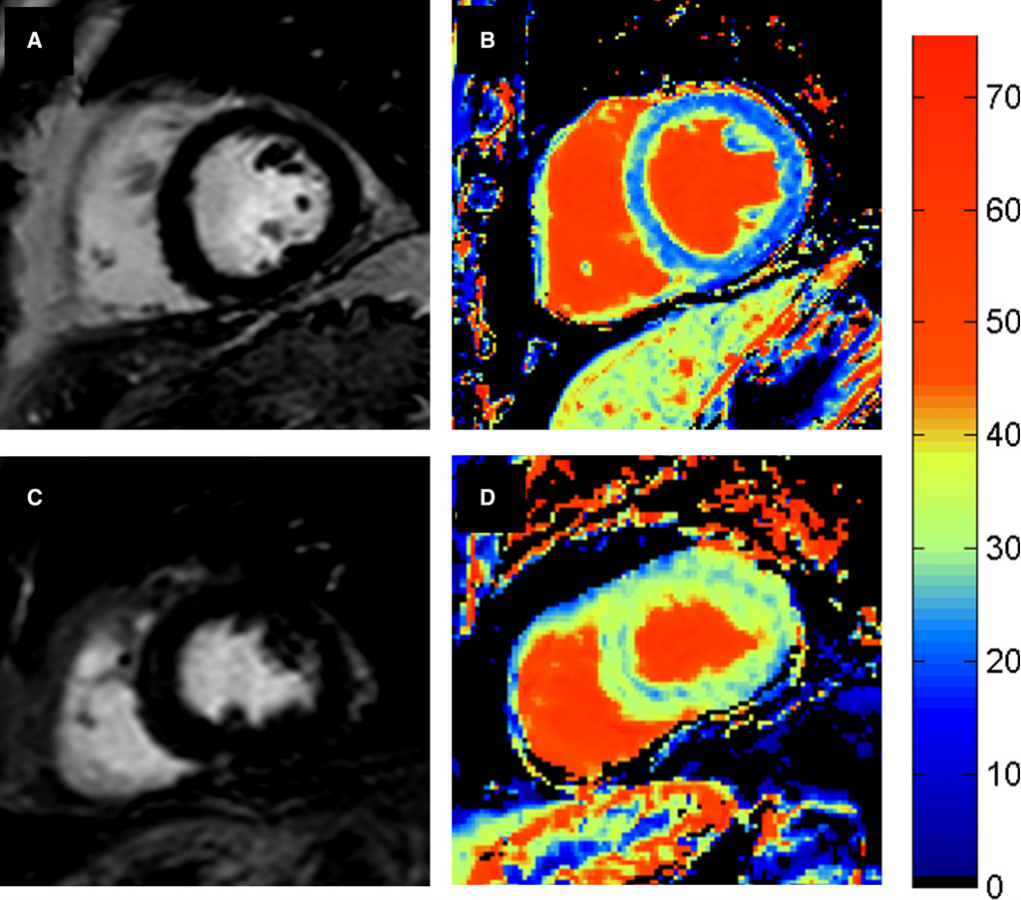
Late gadolinium enhancement (LGE) and extracellular volume (ECV) maps planned in identical imaging planes of an ACR−ve patient (A and B) and ACR+ve patient (C and D). LGE imaging (A and C) showed no focal fibrosis in either patient but ECV mapping (B and D) demonstrated the ECV to be significantly higher in the ACR+ve patient. ACR indicates albumin:creatinine ratio.

T1 mapping could be analyzed in all scans. At baseline, native T1 and ECV were higher in patients with diabetes mellitus than in controls (1242.2±53.9 ms versus 1209.7±47.4 ms, *P*=0.004 and 26.1±3.4% versus 23.3±3.0%, *P*=0.0002). Native T1 and ECV were also higher in ACR+ve than ACR−ve patients (1252.6±66.0 ms versus 1231.72±35.8 ms, *P*=0.05 and 27.2±4.1% versus 25.1±2.9%, *P*=0.004) (Figure 2). A total of 17/100 patients with diabetes mellitus had a subendocardial pattern of LGE in keeping with prior silent MI compared with 0/30 controls, *P*=0.01. There was no difference in the rate of MI between ACR+ve and ACR−ve patients (9/50 and 8/50, respectively; *P*=0.79). Two of 50 ACR+ve and 2/50 ACR−ve patients had clinically significant inducible ischemia on adenosine stress perfusion imaging. None of the controls had inducible ischemia.

There were no significant differences in LV EDV, ESV, mass, EF, or left atrial volume between ACR+ve, ACR−ve, or control (Table 1). Average E**′** measured by echocardiography was lower in ACR+ ve than ACR−ve patients (8.2±1.9 cm/s versus 8.9±1.9 cm/s, *P*=0.04) but there was no significant difference in E/A ratio, average E/E**′,** or S**′**.

There was a trend to more ACR+ve than ACR−ve patients having elevated hs‐cTnT levels (18% versus 4%, *P*=0.05). No difference was seen in the proportion of patients with elevated NT‐proBNP or serum aldosterone levels. Patients with elevated hs‐cTnT had significantly elevated native T1 and ECV (1293.3±80.2 ms and 1235.8±46.5 ms, *P*=0.0001 and 30.3±4.8% versus 25.6±3.2%, *P*<0.0001, respectively). In patients with elevated NT‐proBNP, ECV was higher (29.3±4.5% versus 25.7±3.4%, *P*=0.002) but the difference in native T1 did not reach significance (1285.6±79.6 ms versus 1236.8±47.8 ms, *P*=0.07).

### RAAS Inhibition

Of the 50 ACR+ve patients recruited, 40 returned for follow‐up after 1 year (5 not willing to have the follow‐up scan, 4 lost to follow‐up, and 1 died; Figure 1). Of the remaining 40 patients, 10 were not taking an ACE inhibitor or angiotensin receptor blocker (patient compliance 5, hypotension 2, renal dysfunction 1, hyperkalemia 1, planning pregnancy 1). Therefore, 30 patients completed repeat imaging after a year treatment with RAAS inhibition (median 369, interquartile range 358–385 days). RAAS inhibition was prescribed according to clinician preference and patient tolerance and included ramipril in the majority (23 patients), perindopril in one patient, losartan in 3, candesartan in 2, and irbesartan in a single patient. The final dose was equivalent to ramipril 4.6±2.9 mg.^36^, ^37^

Treatment with RAAS inhibition was associated with a significant decrease in serum aldosterone level (337.0±192.7 to 244.3±137 pmol/L, *P*=0.03) but the change in 24‐hour blood pressure was not statistically significant (Table 2).

**Table 2:** Clinical and Imaging Parameters Pre and Post 1 Year of Treatment with RAAS Inhibition

	ACR+ve Pre RAAS Inhibition	ACR+ve 1 Y Post RAAS Inhibition	Mean Change (95% CI)	*P* Value
N	30	30		
Age (y)	62.9±11.5			
Male sex, n (%)	26 (87)			
Body mass index, kg/m^2^	29.9±4.2	29.8±4.4	−0.04 (−0.57 to 0.48)	0.87
Duration of diabetes mellitus (y)	5.0±4.2			
HbA1c, mmol/mol	60.8±20.0	62.0±15.5	1.7 (−3.5 to 6.9)	0.64
Mean 24‐h systolic BP, mm Hg	136±19	132±20	−2.9 (−11.1 to 5.3)	0.67
Mean 24‐h diastolic BP, mm Hg	73±11	71±11	−2.5 (−5.3 to 0.3)	0.55
Smoking	6 (20)			
LV EDV, mL	148.4±38.7	138.9±37.3	−9.5 (−14.8 to −4.3)	0.001^a^
LV EDV index, mL/m^2^	72.3±16.2	67.6±15.9	−4.7 (−7.3 to −2.1)	0.001^a^
LV ESV, mL	62.4±27.5	55.4±25.1	−7.0 (−11.5 to −2.5)	0.003^a^
Ejection fraction, %	59.3±7.8	61.5±8.7	2.2 (0.2 to 4.3)	0.03^a^
LV mass, g	111.1±27.4	112.7±54.6	1.6 (−1.5 to 4.7)	0.30
LV mass index, g/m^2^	54.0±10.8	54.6±11.3	0.6 (−1.0 to 2.2)	0.46
Mass/end diastolic volume, g/mL	0.76±0.13	0.83±0.28	0.07 (0.03 to 0.11)	0.002^a^
Left atrial volume, mL	91.3±25.8	89.3±28.0	−2.7 (−9.2 to 3.9)	0.41
Left atrial volume index, mL/m^2^	44.5±11.0	43.5±12.2	−1.3 (−4.4 to 1.7)	0.38
Native T1, ms	1243.0±58.5	1251.4±40.9	8.3 (−13.4 to 30.1)	0.44
Extracellular volume, %	26.5±3.6	25.2±3.1	−1.3 (−2.3 to −0.3)	0.01^a^
Evidence of prior myocardial infarction, n (%)	8 (27)	9 (30)		0.50
Serum aldosterone, pmol/L	337.0±192.7	244.3±137.4	−92.7 (−176.2 to −9.1)	0.03^a^
hs‐cTnT ≥14 ng/L, n (%)	6 (20)	8 (27)		0.76
NT‐proBNP ≥125 ng/L, n (%)	4 (13)	7 (23)		0.51
E/A ratio	0.82±0.35	0.84±0.27	0.02 (−0.10 to 0.14)	0.71
E**′** average, cm/s	8.1±1.7	8.1±2.4	0.04 (−0.65 to 0.74)	0.90
E/E**′** average	7.1±1.5	7.4±2.4	0.30 (−0.54 to 1.13)	0.48
S**′** average, cm/s	9.2±2.0	9.4±1.9	0.1 (−0.7 to 1.0)	0.72

ACE indicates angiotensin‐converting enzyme; ACR, albumin:creatinine ratio; EDV, end diastolic volume; ESV, end systolic volume; HbA1c, hemoglobin A1c; hs‐cTnT, high‐sensitivity cardiac troponin T; LV, left ventricle; NT‐proBNP, amino terminal B type natriuretic peptide; RAAS, renin‐angiotensin‐aldosterone system.

a *P*≤0.05.

### Effect of ACE Inhibition on Cardiac Structure, Function, and Composition

After 1‐year treatment with RAAS inhibition 14/30 (47%) had a decrease in ACR to the extent they would no longer be considered ACR+ve. RAAS inhibition was associated with a decrease in ECV (26.5±3.6 to 25.2±3.1%, *P*=0.01) but not native T1 (1243.0±58.5 to 1251.4±40.9 ms, *P*=0.44). There was also a significant decrease in LV EDV (148.4±38.7 to 138.9±37.3 mL, *P*=0.001) and an associated increase in LV EF (59.3±7.8 to 61.5±8.7%, *P*=0.03) but there was no significant change in LV mass or left atrial volume. E/A ratio, E**′**, E/E**′,** or S**′** measured by echocardiography were not altered by treatment with RAAS inhibition. On repeat biomarker assessment the proportion of patients with elevated hs‐cTnT or NT‐proBNP was not altered by RAAS inhibition.

## Discussion

At baseline, asymptomatic patients with type 2 diabetes mellitus and persistent microalbuminuria had increased cardiac native T1 and ECV, reduced E**′** measured by echocardiography, and elevated hs‐cTnT. There was no difference in the rates of myocardial ischemia or infarction, suggesting that the reported differences are because of a process of microvascular changes and myocardial extracellular fibrosis rather than occult CAD. One‐year treatment with RAAS inhibition was associated with a decrease in both ECV and improvement in LV EF. This did not appear to be mediated by blood pressure reduction, which was not significantly lower on treatment.

### Myocardial Tissue Composition

We report, in keeping with previous findings, that type 2 diabetes mellitus is associated with increased ECV compared with healthy controls.^19^, ^38^, ^39^ Additionally, we demonstrate that ACR+ve patients have higher ECV than ACR−ve patients and that elevated ECV in these patients is reversible with RAAS inhibition. These observations suggest that diffuse cardiac fibrosis could mediate heart failure risk in patients with persistent microalbuminuria.^11^, ^12^ We consciously recruited lower risk patients than previous studies (excluding those with being treated with RAAS inhibition or insulin), which may in part explain why ECV in diabetes mellitus patients in the present study were not as high as previous studies using similar methods.^21^

Previous studies have demonstrated that in asymptomatic patients without known CAD, increased urinary aldosterone levels and angiotensin II–mediated aldosterone increase are associated with increased ECV.^20^ Furthermore, in a retrospective cross‐sectional study of patients undergoing CMR for clinical purposes, the use of RAAS‐inhibiting medication was associated with lower ECV.^21^ Ours is the first prospective study to show that ECV can be altered in patients with diabetes mellitus and adds further weight to the hypothesis that increased RAAS activity mediates diffuse fibrosis, consequently increasing heart failure risk, and that RAAS inhibition may be a potential therapeutic intervention to ameliorate this risk.

Our findings contrast in part to those by Jellis et al in which patients with diabetes mellitus treated with spironolactone failed to show a reduction in ECV.^40^ This result may be explained by the high use of ACE inhibitors at baseline, exclusion of patients without diastolic dysfunction, and the use of a now‐outdated pulse sequence for the estimation of myocardial ECV. Alternatively, it is possible that ACE inhibitors are more effective than aldosterone blockers at ameliorating heart failure risk in this population. On the other hand, our results are in keeping with those of Garg et al who have reported that microvascular function measured by positron emission tomography improves by RAAS inhibition, using spironolactone in asymptomatic patients with type 2 diabetes mellitus.^41^

### Coronary Artery Disease

The patients studied were all asymptomatic with no prior cardiac disease. However, there was still a relatively high proportion of patients with diabetes mellitus who had prior MI (17%). This rate at baseline was already comparable with the 15.5% Framingham predicted risk of developing coronary disease over the subsequent 10 years. This result is also in keeping with previous studies of patients with type 2 diabetes mellitus, although it should be noted that patients in these studies were not necessarily asymptomatic.^24^, ^25^ Microalbuminuria has previously been associated with excess risk of myocardial infarction.^9^, ^12^ However, in the present study the rate of MI was not influenced by ACR status, suggesting that it is not the mechanism mediating increased heart failure risk in ACR+ve patients.

Conversely, the rate of inducible ischemia detected on adenosine stress CMR was only 4%, which is lower than comparable studies using single‐photon emission computed tomography.^42^ This may reflect the low risk of patients in the present study and the different sensitivity and specificity of adenosine stress perfusion CMR and single‐photon emission computed tomography to detect stable CAD.^34^

### Left Ventricular Function

Diastolic dysfunction was a common finding in our study population (both ACR+ve and ACR−ve had E/A reversal), in keeping with previous studies.^17^, ^18^ However, ACR+ve patients had a lower E**′,** suggesting more advanced diastolic dysfunction. Previous studies have suggested that more severe diastolic dysfunction in diabetes mellitus predicts those who are likely to go on to develop clinically important heart failure.^43^ We have also identified that diabetes mellitus is associated with concentric remodeling of the left ventricle,^16^, ^44^ although there were no differences between ACR+ve and ACR−ve patients.

Treatment with RAAS inhibition was associated with a modest improvement in systolic function, although there were no significant changes in diastolic function measured by echocardiography. This is in keeping with the observation that RAAS inhibition is a much better treatment for heart failure with reduced EF than with preserved EF and diastolic dysfunction.^45^

Interestingly, RAAS inhibition was not associated with a decrease in LV mass. It is well recognized that there is a strong correlation between blood pressure and LV mass.^16^, ^46^ The mean dose of RAAS inhibition equivalent to ramipril was 4.6 mg, which reflects the difficulties in clinical practice of uptitration, and it is possible that higher doses of RAAS inhibition would be associated with a greater reduction of blood pressure and consequently LV mass.

### Biomarkers

Microalbuminuria has been used for some time as a screening biomarker to identify patients with diabetes mellitus with increased cardiovascular risk,^30^ and current guidelines suggest that those with persistent microalbuminuria should receive intensive cardiac preventative medication (although the cut‐offs used to diagnose microalbuminuria vary between guidelines).^47^ However, though ACR is a powerful screening tool, there can be wide variations between repeated measurements and ACR can be falsely elevated in the presence of glycosuria, infection, strenuous exercise, fever, and menstruation.^48^

Patients with type 2 diabetes mellitus have elevated levels of both hs‐cTnT and NT‐proBNP, which appear to be predictive of adverse cardiovascular events including heart failure.^27^, ^28^, ^29^ However, previous studies that have demonstrated their prognostic utility have not included comprehensive cardiac imaging and the exact mechanism of the processes leading to myocardial injury and biomarker release was not fully understood. We have demonstrated that in ACR+ve patients both hs‐cTnT and ECV are elevated, suggesting that they are both markers of a diffuse cardiac fibrotic process rather than occult CAD. Future randomized studies are needed to establish whether ECV, hs‐cTnT, or diastolic dysfunction themselves or the associated adverse prognosis can be altered by intervention.

### Limitations

This study was a nonblinded observational study, and the results need to be confirmed in a larger blinded randomized cohort. Furthermore, the study was not powered to investigate the prevalence of silent CAD or heart failure end points but did include multiple surrogate imaging end points. We have not carried out coronary angiography because this invasive procedure would not be ethically appropriate in asymptomatic low‐risk patients. However, stress perfusion imaging excluded significant silent ischemia in these patients and therefore CAD is unlikely to be responsible for our findings. For the same reasons, we did not carry out myocardial biopsy to confirm increased extracellular matrix histologically. However, ECV measured by CMR has been validated against histology in other disease processes^33^ and in animal models of diabetes mellitus.^49^ We did not perform echocardiography or biomarker assessment on the healthy controls, although normal ranges exist for these parameters.

## Conclusions

Asymptomatic patients with type 2 diabetes mellitus and persistent microalbuminuria have several markers suggestive of diffuse cardiac fibrosis including elevated ECV, hs‐cTnT, and diastolic dysfunction. The prevalence of silent myocardial ischemia or infarction was not influenced by ACR status. Treatment with an ACE inhibitor was associated with improvement in LV EF and ECV regression. These findings suggest that increased risk in this patient group is mediated by subclinical changes in tissue structure and function rather than occult CAD, which may have implications for the management of these patients.

## Author Contributions

Guarantors of integrity of entire study Swoboda, Plein; study concepts/study design or data acquisition or data analysis/interpretation, all authors; manuscript drafting or manuscript revision for important intellectual content, all authors; approval of final version of submitted manuscript, all authors; literature research, Swoboda, Plein; statistical analysis, Swoboda, and manuscript editing, all authors.

## Sources of Funding

Swoboda (FS/12/88/29901) and Plein (FS/1062/28409) are funded by British Heart Foundation fellowships. This study was supported by the National Institute for Health Research Leeds Clinical Research Facility. The views expressed are those of the author(s) and not necessarily those of the NHS, NIHR, or the Department of Health.

## Disclosures

Witte and Kearney have received research funding from Medtronic UK. Other authors have no disclosures.
